# Fine-scale population structure in five rural populations from the Spanish Eastern Pyrenees using high-coverage whole-genome sequence data

**DOI:** 10.1038/s41431-021-00875-0

**Published:** 2021-04-09

**Authors:** Iago Maceda, Miguel Martín Álvarez, Georgios Athanasiadis, Raúl Tonda, Jordi Camps, Sergi Beltran, Agustí Camps, Jordi Fàbrega, Josefina Felisart, Joan Grané, José Luis Remón, Jordi Serra, Pedro Moral, Oscar Lao

**Affiliations:** 1 CNAG-CRG, Centre for Genomic Regulation, C/ Baldiri i Reixach 4, 08028 Barcelona, Spain; 2 Barcelona Institute of Science and Technology (BIST), Barcelona, Spain; 3 Universitat Pompeu Fabra (UPF), Barcelona, Spain; 4 Department of Evolutionary Biology, Ecology and Environmental Sciences, Biodiversity Research Institute, Faculty of Biology, University of Barcelona, 08028 Barcelona, Spain; 5 Institute of Biological Psychiatry, Mental Health Services, Sct. Hans, Roskilde, Denmark; 6 Hospital Sant Bernabé, Ctra. de Ribes 47, 08600 Berga, Barcelona Spain; 7 Fundació Sant Hospital La Seu d’Urgell, Pg. Joan Brudieu 8, La Seu d’Urgell, Lleida Spain; 8 Hospital d’Olot i Comarcal de la Garrotxa, Avda. Països Catalans 86, 17800 Olot, Girona Spain; 9 Hospital de Campdevànol, Ctra. de Gombrèn 20, 17530 Campdevànol, Girona Spain; 10 Servei d’atenció primaria Lleida Nord, Gerència Territorial Alt Pirineu i Aran, Institut Català de la Salut, C/ Sant Jordi 13, 25620 Tremp, Lleida Spain; 11 Laboratori d’Anàlisis Clíniques, Hospital Comarcal del Pallars, C/ Pau Casals 5, 25500 Tremp, Lleida Spain

**Keywords:** Genetic variation, Inbreeding

## Abstract

The area of the Spanish Pyrenees is particularly interesting for studying the demographic dynamics of European rural areas given its orography, the main traditional rural condition of its population and the reported higher patterns of consanguinity of the region. Previous genetic studies suggest a gradient of genetic continuity of the area in the West to East axis. However, it has been shown that micro-population substructure can be detected when considering high-quality NGS data and using spatial explicit methods. In this work, we have analyzed the genome of 30 individuals sequenced at 40× from five different valleys in the Spanish Eastern Pyrenees (SEP) separated by less than 140 km along a west to east axis. Using haplotype-based methods and spatial analyses, we have been able to detect micro-population substructure within SEP not seen in previous studies. Linkage disequilibrium and autozygosity analyses suggest that the SEP populations show diverse demographic histories. In agreement with these results, demographic modeling by means of ABC-DL identify heterogeneity in their effective population sizes despite of their close geographic proximity, and suggests that the population substructure within SEP could have appeared around 2500 years ago. Overall, these results suggest that each rural population of the Pyrenees could represent a unique entity.

## Introduction

In Europe, the transition from a rural to an urban world has been mainly triggered by the industrial revolution. This event promoted large-scale, differentiated and coordinated activities that were better accomplished by urban communities [1], which caused population movements from rural areas to urban cities [2]. From a practical point of view, the division in rural and urban areas has implications for health [3], as well as for generating genetic isolates explaining the predisposition to rare diseases [4].

In Spain, rural areas comprised 68% of the total Spanish population by 1900 [5], and similarly to other rural European regions, they have been intensively depopulated with massive migratory movements towards industrialized urban areas [6]. Spanish rural areas are traditionally characterized by a low-demographic density and a high number of small municipalities that may have experienced isolation for generations [7]. This situation has been suggested as a main factor for explaining the higher levels of consanguinity of Spain compared to other European countries [8]. From a temporal point of view, the level of consanguinity in urban, and particularly, rural Spanish areas reached its maximum between the end of the 19th century and 1929 [8].

The main force explaining the higher inbreeding coefficients in Spanish rural areas compared to urban areas is geography [9]. In particular, islands and high mountains, as well as altitude within a valley, have been reported as the most effective geographic barriers increasing the levels of inbreeding in Spain [9]. In this context, the rural population of the Spanish Eastern Pyrenees (SEP) has been suggested as a particularly interesting system for understanding the demographic dynamics of traditional Spanish rural areas [10]. The Pyrenees is a mountain chain of a complex orography spanning 430 km West to East and separating the North of the Iberian Peninsula from the rest of Europe [11]. From a demographic point of view, the area reached its maximum-recorded population peak in 1860 and it has been intensively depopulated since then [12].

Studies using classical markers, such as blood markers, proteins, and HLA antigens [13] did not detect genetic barriers within the Spanish Pyrenees but a strong West to East gradient. Others using immunoglobulin data [14] did not replicate these results but proposed that the observed patterns of diversity are better explained by microgeographic differentiation. One Y chromosome study detected a subtle degree of substructure in the whole Spanish Pyrenees mountain range [15]. The most recent study considered autosomal microarray data and it did not find any genetic difference with other Iberian samples nor detected signals of excess of autozygosity compatible with endogamous practices in the region [16]. Overall, the discrepancies among these studies suggest that, if the orography of the Pyrenees has shaped the genetic diversity of the rural human populations living within this mountain chain, a much deeper characterization of their genetic variation is required to detect it.

In the present study, we have characterized the genetic variation of the SEP rural population from five regions (Pallars (P), Alt Urgell (U), Berguedà (B), Ripollès (R), and Garrotxa (G)) separated by less than 140 km along a west to east axis, making use, for the first time, of high-coverage whole-genome sequencing (WGS) data. This allowed us the use of powerful haplotype-based methods (such as Chromopainter/fineSTRUCTURE [17] or GLOBETROTTER [18]), revealing genetic differences between close groups. Likewise, it ensured an unbiased characterization of the allele frequency spectrum, something necessary in demographic modeling.

## Material and methods

### Datasets description

In total, five regions corresponding to the political separations established by the government from SEP were sampled (Garrotxa, Ripollès, Berguedà, Alt Urgell and Pallars), with six samples per region. All sampled individuals were born and had all their grandparents born in the same sampled region. This dataset represents the oldest extract of the population, with an average age of ~76 years and equal proportions of both sexes (see Supplementary Table 1). Therefore, this sample is effectively unaffected by the demographic changes occurred during the 20th century. All subjects signed an informed consent and the study had the approval of the Ethics Committee of the University of Barcelona. Each individual was whole-genome sequenced using standard Illumina (San Diego, California, USA) paired-ends sequencing technology with a read-length of 150 bp with an average sequencing coverage of ~40×. Single Nucleotide Variant (SNV) calling used GATK HaplotypeCaller v3.6 [19], using the default settings according to the GATK Handbook v3.6 [20], with hs37d5 as the reference assembly, using all samples jointly. For an overview on the data cleaning steps, see Supplementary Information. The final dataset contained 29 individuals and 9,309,056 SNVs. Data are available upon request to the corresponding author.

To make comparisons with other European populations, we used samples geographically classified as “West Eurasian” from the Simons Genome Diversity Project (SGDP) [21] (Supplementary Table 1). After the merging with the SEP dataset, we applied the same quality control as conducted with SEP. The SEP-SGDP dataset contains a total of 104 individuals and 5,388,964 SNVs.

Spanish exome data from [22] was accessed from EGA (accession number: EGAS00001000938). Data were downloaded as FASTQ files and mapped. SNVs were called following the same procedure used in the SEP samples. We used the same data cleaning procedures as conducted with SEP. The resulting dataset has a total of 288 individuals and 239,349 SNVs. To make a comparison between our dataset and the one presented in [16], we ascertained the shared SNVs between the two datasets, namely ~231 K SNVs.

### Analyses

#### Detection of population substructure of SEP at a macro and microgeographic scale

A metric multidimensional scaling (MDS) analysis, also called classical multidimensional scaling [23] on an identical by state (IBS) matrix between pairs of individuals (‘1-ibs’ function of PLINK [24]) was carried out to summarize the genomic relationships within SEP as well as with SGDP samples. SEP-SGDP data were phased with SHAPEIT2 [25] using default values and a publicly available genetic map based on the 1000 Genomes Project phase 3 database [26]. Phased data were used in Chromopainter/fineSTRUCTURE [17] to identify fine-population substructure. The haplotype lengths matrices from Chromopainter/fineSTRUCTURE were analyzed with GLOBETROTTER [18]. Finally, we repeated the haplotype-based analyses with SEP individuals in order to detect fine-scale population substructure in the area.

#### Identification of genetic barriers and differential migration rates

In order to identify genetic barriers, we developed an algorithm that models the shared co-ancestry matrix from Chromopainter/fineSTRUCTURE in terms of anisotropy within each geographic group (see Supplementary Information). In parallel, we used the Estimated Effective Migration Surfaces (EEMS) algorithm to have an estimate of migration rates between the individuals of SEP dataset, [27]. The algorithm was run using the default parameters and a total of 1000 demes to conform the surface on which to situate the individuals. From a practical point of view, we set the number of demes to 1000, so all the samples could be positioned in individual demes given their close geographical proximity.

#### Estimation of the effective population sizes and time of split of SEP populations

**Fig. 1:**
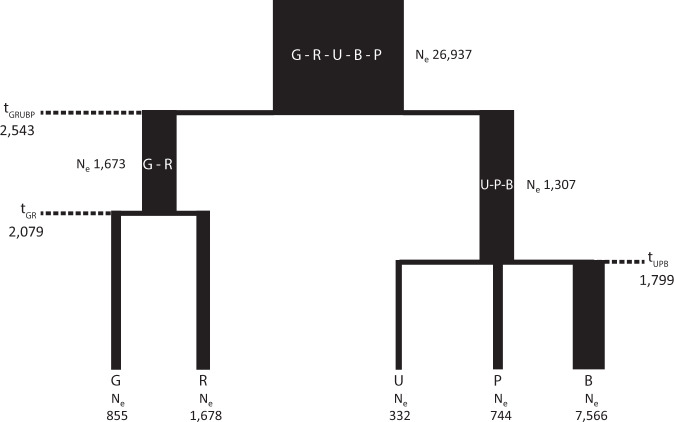
Representation of the demography used in the ABC_DL approach. All numbers represent the median of the posterior distribution. Migration between actual populations is included in the demographic model, but not plotted in this graphic. Times of split represent years before present assuming 29 years by generation [37]. (G: Garrotxa; R: Ripollès; U: Alt Urgell; B: Berguedà; P: Pallars; GRUBP: meta-population of the Pyrenees; GR: meta-population of the subgroup formed by Garrotxa and Ripollès; UPB: meta-population of the subgroup formed by Alt Urgell, Pallars, and Berguedà; t_GRUBP_: time of split of the meta-population in the two sub populations; t_GR_: time of split of the meta-population for the Garrotxa and Ripollès subgroup; t_UPB_: time of split of the meta-population for the Alt Urgell, Pallars, and Berguedà).

To estimate the effective population size and the time of split of the SEP regions, we modeled their demographic history using a simple demographic model (see Fig. 1). We used an ABC approach coupled to deep learning (ABC-DL) [28] to estimate the posterior distribution of the different parameters of the model (see Table 1 for the prior distributions, Supplementary Fig. 1 for density distribution of prior and posterior probabilities of the parameters and Supplementary Information for details).

**Table 1:** Prior distributions, mean, median, half mode range, 95% credible interval (CI 2.5–97.5%) and 89% Highest Density Interval (HDI) of the posterior distributions for the effective population size of the meta-population (Ne_GRUBP_), meta-population of the Garrotxa-Ripollès cluster (Ne_GR_), meta-population of the Urgell-Berguedà-Pallars cluster (Ne_UPB_), Garrotxa (Ne_G_), Ripollès (Ne_R_), Urgell (Ne_U_), Berguedà (Ne_B_), and Pallars (Ne_P_) and the time of the split (t_GRUBP_, t_GR_, and t_UBP_) in years before present, assuming a generation time of 29 years [37] estimated using ABC-DL.

	Prior	Posterior						
		Mean	Median	Half range mode	CI 2.5%	CI 97.5%	Low HDI	High HDI
Ne_GRUPB_	U (15000, 40000)	26937.89	27079.55	27189.87	25469.85	27477.40	26258.71	27585.47
Ne_GR_	U (1000, 5000)	1673.31	1586.39	1271.04	1019.46	2832.32	1004.09	2268.51
Ne_UPB_	U (1000, 5000)	1307.32	1248.50	1009.04	1009.96	1943.13	1000.98	1628.88
Ne_G_	U (500, 2500)	855.28	840.17	882.52	523.68	1333.39	500.87	1113.76
Ne_R_	U (500, 2500)	1678.49	1678.87	1544.19	806.70	2452.33	1106.79	2484.24
Ne_U_	U (200, 2000)	332.79	320.11	289.09	207.48	539.60	200.55	445.53
Ne_P_	U (500, 2500)	744.77	719.12	675.06	509.81	1124.30	500.92	966.54
Ne_B_	U (500, 10000)	7566.18	7766.21	9836.68	4054.06	9904.54	5250.46	9988.18
t_GRUBP_	U (2320, 14500)	2543.51	2480.21	2334.04	2333.51	3087.88	2330.91	2812.27
t_GR_	U (870, tGRUPB)	2079.06	1960.69	1554.95	942.59	3834.94	892.55	3053.28
t_UPB_	U (870, tGRUPB)	1799.98	1698.16	1235.55	925.78	3152.49	894.68	2587.97

All prior distributions follow a Uniform distribution (U). Nevertheless, since t_GR_ and t_UBP_ depend on t_GRUBP_, the final shape of these prior distributions is not Uniform.

#### Quantification of linkage disequilibrium and levels of autozygosity in SEP and Spanish exomes samples

The decay of linkage disequilibrium (LD) was estimated for each population with the HR statistic [29]. In order to minimize the effects of frequency-dependence in LD measures [30], LD was computed by averaging the HR between pairs of SNVs showing a similar MAFs (|MAF_SNVa_ − MAF_SNVb_ | < 0.05). Runs of homozygosity (RoH) were quantified by means of two different approaches. For WGS data from SEP, we used the RoH as defined in [31]. For the exomic data, we used the heterozygosity ratio (HetR) [32]. This measure is defined as the ratio of SNVs for which the individual is heterozygote with respect to the number of SNVs for which the individual is homozygote for the non-reference allele. As we have an extremely different sample size between the two sets of datasets (maximum of six and minimum of five individuals in SEP regions, 259 individuals in the SpExomes), we sampled sets of five samples from every region and calculated the HetR for each selected sample. We repeated this sampling a total of 5000 times. A normalized estimate (nHetR) was obtained averaging all the replicates for each sample.

## Results

### Genetic variation of SEP in the European context

In order to describe the genetic relationships of the SEP samples with the European continent, we first performed a MDS analysis. SEP populations cluster with samples from the Iberian Peninsula (Supplementary Fig. 2), following the geographic dependence of the genetic diversity observed for whole Europe in other studies [33]. Complementary to these analyses, we ran a Chromopainter/fineSTRUCTURE analysis with the SEP-SGDP dataset. In agreement with the previous result, the phylogenetic tree (Supplementary Fig. 3) shows all individuals from SEP sharing a private cluster with the Basque samples from the French Western Pyrenees. The GLOBETROTTER analysis (Supplementary Fig. 4) did not identify a differential genomic contribution to SEP from any particular European population from the SGDP dataset, thus suggesting that historical migrations did not influence the population substructure present in the SEP.

### Fine-scale population structure in SEP

**Fig. 2:**
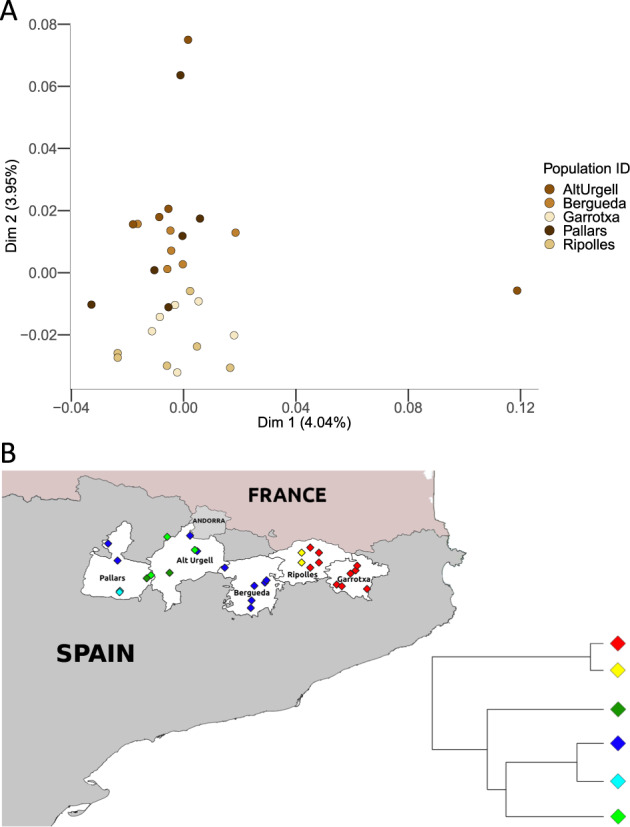
Genetic variation of SEP. **A** Metric MDS of the samples from SEP. **B** Map showing SEP painted accordingly to the cluster they belong to. Ripollès samples are artificially dispersed for the sake of clarity. Simplified fineSTRUCTURE tree of SEP samples, showing six clusters which can be further summarized in two main groups: Garrotxa-Ripollès and Pallars-Alt Urgell-Berguedà.

**Fig. 3:**
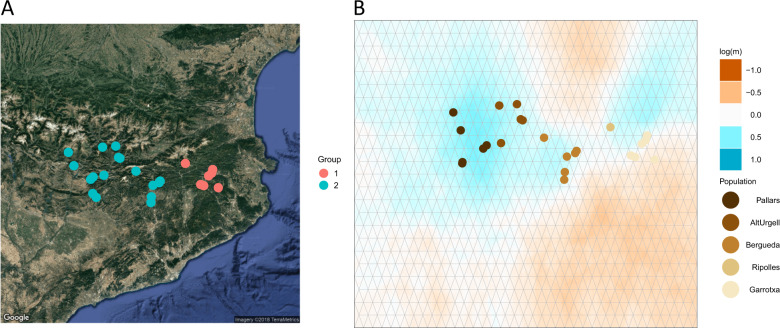
Autozygosity, inbreeding, and LD in SEP. **A** Genetic barrier between Garrotxa-Ripollès (red dots) and Pallars-Alt Urgell-Berguedà (blue dots) identified by an algorithm that models the genetic variation present in the data in terms of anisotropy and genetic barriers. **B** EEMS result also state a migration barrier between Garrotxa-Ripollès and Pallars-Alt Urgell-Berguedà.

We wondered if such structure would extend at a microgeographic level in SEP. We repeated the MDS and Chromopainter/fineSTRUCTURE analyses considering the SEP samples alone. As shown in Fig. 2A, the first two dimensions mimic the geographic sampling location (correlation in a symmetric Procrustes rotation = 0.35 (*p* value = 0.033) after 99,999 permutations), mainly distributing the SEP samples in the longitudinal axis in the second dimension. Chromopainter/fineSTRUCTURE also identified two main clusters that split the west to east axis (Fig. 2B). These clusters correspond to the regions of Garrotxa and Ripollès (GR) and the regions of Pallars, Alt Urgell and Berguedà (PUB). In addition to these two main clusters, Chromopainter/fineSTRUCTURE identified further population substructure within the two subgroups. However, these additional groups did not show a clear geographic pattern. We wondered if this result could reflect different patterns of spatial anisotropy in our data. We applied an algorithm that describes the genetic relationship between individuals in terms of genetic barriers and different patterns of anisotropy between groups (see Supplementary Information). Our result for two groups (Fig. 3A) detected a genetic barrier between GR and PUB. In order to confirm this identified genetic barrier, we ran EEMS in the SEP dataset. As shown in Fig. 3B, EEMS identified a migration depletion compared to the rest of the geographic area between the same set of regions previously detected by Chromopainter/fineSTRUCTURE analysis and apparently separated by genetic boundaries. This previously undetected population substructure compared to microarray data [16] could reflect differences in WGS vs. array-based data and/or the applied methodology. Using the SNVs that are common with those used in [16], Chromopainter/fineSTRUCTURE failed to detect geographic clusters (see Supplementary Fig. 5). Overall, these results suggest the presence of micro-population substructure in SEP that requires WGS to be detected.

### Autozygosity, inbreeding, and LD in SEP compared to Spanish exomes

**Fig. 4:**
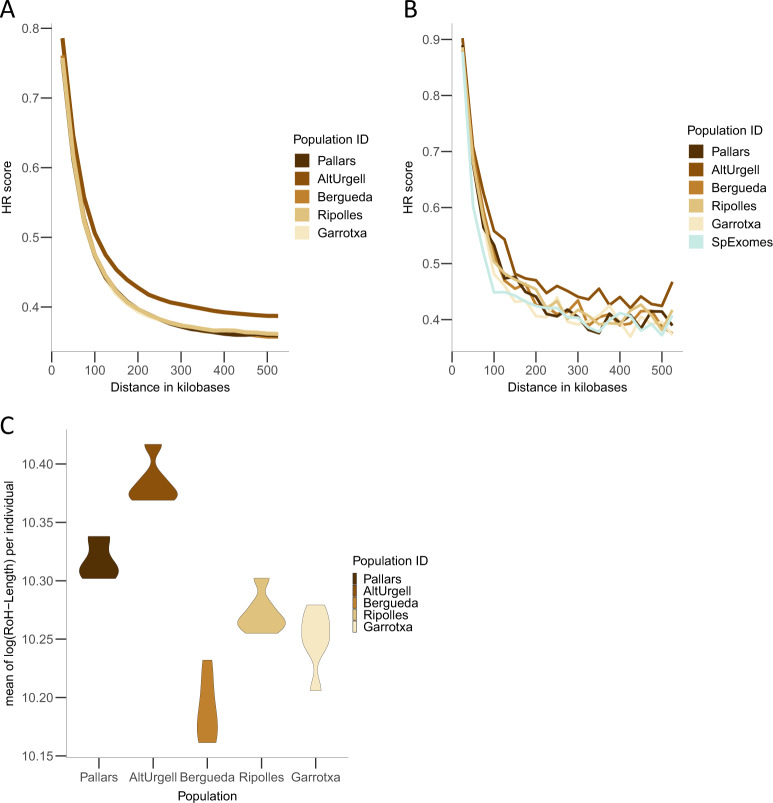
Decay of LD and ROH and HetR of SEP and SpExomes samples. **A** LD decay in SEP samples using WGS. **B** LD decay of SEP samples with the Spanish exomes dataset using exome sequencing data. **C** Violin plot of the total amount of homozygous fragments in each SEP individual using WGS.

We analyzed the patterns of LD by means of HR, using the genetic variation present in the whole-genome of the SEP populations. We observed differences between the SEP populations in the decay of LD heterogeneity (Fig. 4A). In particular, the Alt Urgell region showed more LD than others. We wondered whether the observed pattern was specific of SEP, or if it was particular to the general Spanish population. We repeated the LD analyses on the exome using the Spanish exomes dataset. First, we observed that the dataset (WGS vs. exome) did not influence the decay of the HR score in SEP populations (Supplementary Fig. 6). When comparing SEP and Spanish exomes, we observed that all regions have higher HR scores than Spanish exomes and, again, that Alt Urgell is the one that has the highest HR score among all the populations sampled (Fig. 4B).

We wondered whether these results would be in agreement with the reduction of genetic diversity due to a traditionally low-demographic density and endogamy. We found that not all the populations showed the same amount of RoHs per individual (Kruskal–Wallis test *P* value = 4.413e−05). Out of the five considered populations, Alt Urgell was the one with the longest RoHs and Berguedà with the shortest ones (Fig. 4C). When comparing SEP populations with the SpExomes dataset, Alt Urgell has the lowest nHetR of all the SEP populations and within the nHetR of the SpExomes (Supplementary Fig. 7).

Therefore, all the results suggest that the rural populations of the Pyrenees show particular demographic histories compared with the general Spanish population as well as between them, despite their close geographic proximity.

### Demographic history of SEP

We modeled the demography of the SEP populations by means of ABC-DL (see “Material and Methods” section). Following the observed population structure in previous analyses, we assumed a demographic topology that clustered PUB at one branch, and GR at the other. The ABC-DL analysis suggests that the overall observed population substructure among SEP regions appeared around 2500 years ago (95% CI from 2333.51 to 3087.88 years ago), independently of which centrality statistic was used (Table 1). According to ABC-DL, the split between GR would have taken place around 1554.95–2079.06 years ago depending on the centrality statistic (95% CI from 942.59 to 3834.94 years ago). The estimated split time within the PUB cluster was 1235.55–1799.98 years ago (95% CI from 925.78 to 3152.49 years ago). However, both the 95% credible interval range and the 89% high-density interval of the three posterior distributions is quite broad and overlap, compatible with a similar time of genesis of the overall population substructure of the area. Interestingly, we observed differences in the effective population size of the five SEP regions. Alt Urgell region showed the smallest effective population size (mean of the posterior distribution = 332.79; 95% CI ranging from 207.48 to 539.60 chromosomes); in contrast, the mean of the estimated effective population size of Berguedà was 7566.18 chromosomes (95% CI from 4054.06 to 9904.54). These results agree with the different decay of LD and different RoHs patterns in Alt Urgell compared to the other SEP populations.

## Discussion

In agreement with previous results based on non-NGS data [16, 33], our MDS analyses place the SEP individuals within the South Western context of the genomic diversity within Europe and, particularly, within the Iberian samples of the SGDP dataset. Within SEP, we recover the west to east axis of genetic diversity in the Pyrenees initially reported using classical markers, which has been explained in terms of the original peopling of the area [13]. More interestingly, our analyses detected the presence of ultra-fine-geographic differentiation across the five SEP populations when using haplotype-based data and Chromopainter/fineSTRUCTURE. Similar levels of ultra-fine genetic differentiation have been observed in some rural regions of Galicia when using Chromopainter/fineSTRUCTURE [34]. Nevertheless, in our study, we observed that this fine-population substructure can only be detected by using WGS. The two clusters identified by Chromopainter/fineSTRUCTURE show a marked geographical component, as estimated by a spatial model that includes geographic barriers and anisotropy, and independently replicated by EEMS. The orography between the two clusters cannot explain this genetic differentiation, as within the PUB cluster there are greater orographic phenomena than between PUB and GR. Furthermore, the presence in Alt Urgell and Pallars of the genetic component characteristic of Berguedà (in blue in Fig. 2B), but not in Ripollès, which is geographically closer to Berguedà, suggests the presence of complex historical demographic processes within SEP on top of the orography.

The absence of differences between regions with respect to their patterns of Western Eurasian ancestry, as shown in the GLOBETROTTER results, suggests that geographic isolation within SEP is likely the cause of the identified substructure. This isolation should have appeared after major migrations into the region, or these had a very limited impact in the genetic makeup of SEP. In particular, it has been claimed that fine genetic variation has been shaped in Spain by linguistic and geopolitical boundaries at the time of Muslim rule in Spain [34]. However, the Roman and Visigoth people only represented a 2.2–4.4% of the SEP population, while the Islamic conquest of this region lasted only 80 years [35]. In this line, ABC-DL suggests that the population structure observed in SEP originated around 2500 years ago. Furthermore, the low effective population sizes inferred from genomic data support genetic isolation as the main factor for explaining the geographic structure. However, it is interesting to notice the large heterogeneity in the estimates of the effective population size given the geographic proximity of the SEP populations. These estimations are in agreement with the estimated levels of autozygosity and LD. Therefore, despite the short distances between populations, the particular demographic histories of the different villages played a role in shaping the genomic landscape of the regions. In fact, these counties traditionally shared a rural lifestyle but considered different methods of subsistence depending on their geographic location and period [35]. For example, by the end of the XIX century, the economy of Berguedà focused on the exploitation of natural resources (in the upper part) and textile (in the lower part). This type of economy granted a railway to the county connecting it to the most industrialized part of Catalonia by 1914 [36] which could have favored more admixture with populations from other regions.

In this work, we have described the genetic variation of five rural villages from the SEP through the analysis of high-coverage WGS data accompanied by detailed genealogical information. Our results suggest that geographically close SEP villages could show particular demographic histories. However, further analyses will be required to study if the observed pattern extends to other geographic regions of the Pyrenees, at both the Spanish and French side, as well as to other Spanish areas.

## Supplementary information

Supplementary material
