# Supplementary material for: Fine-scale population structure in five rural populations from the Spanish Eastern Pyrenees using high-coverage whole-genome sequence data
Source: Eur J Hum Genet. 2021 Apr 9;29(10):1557–65. doi: 10.1038/s41431-021-00875-0 (PMC8484665; doi:10.1038/s41431-021-00875-0)
Supplement: Supplementary file 1 — Supplementary material [file 41431_2021_875_MOESM1_ESM.pdf]

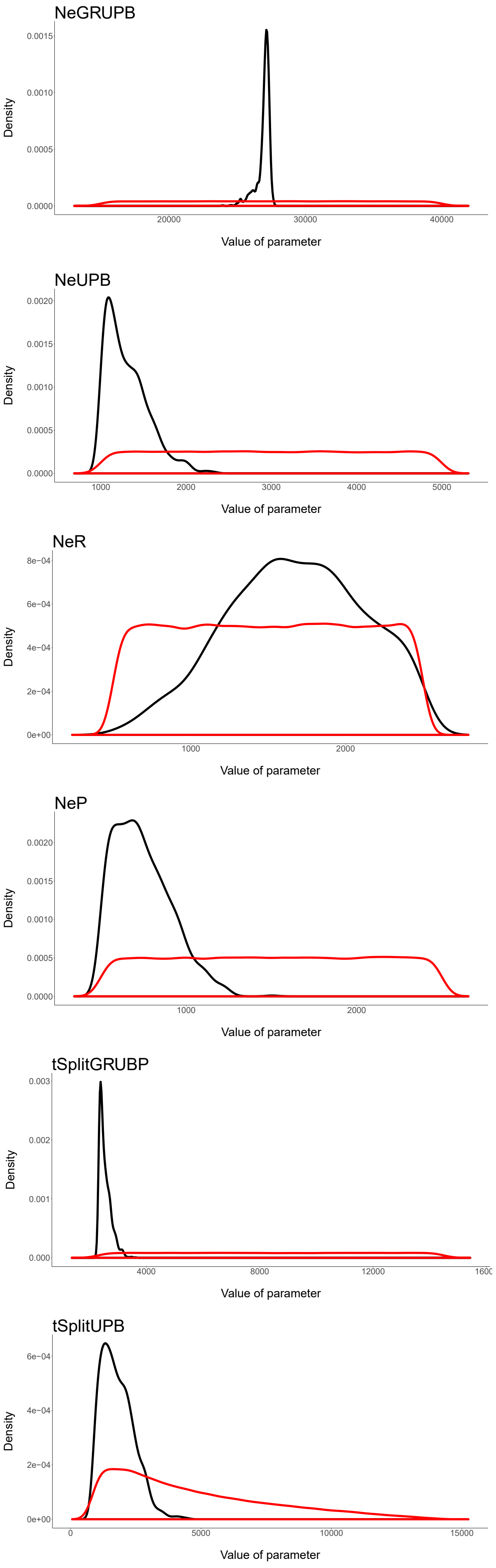

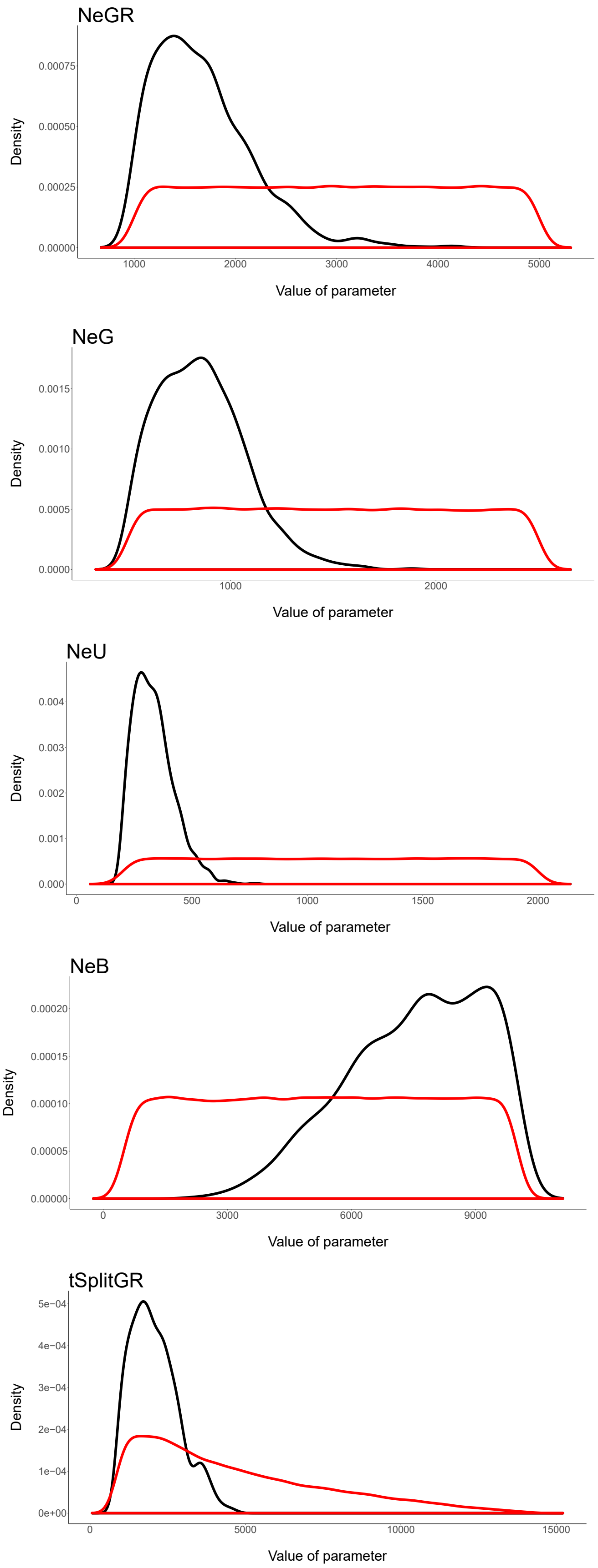

Supplementary Figure 1. Prior and posterior distribution for the parameters of ABC-DL. Prior distribution is colored red; posterior distribution is colored black. Times of split are ploted as years before present.

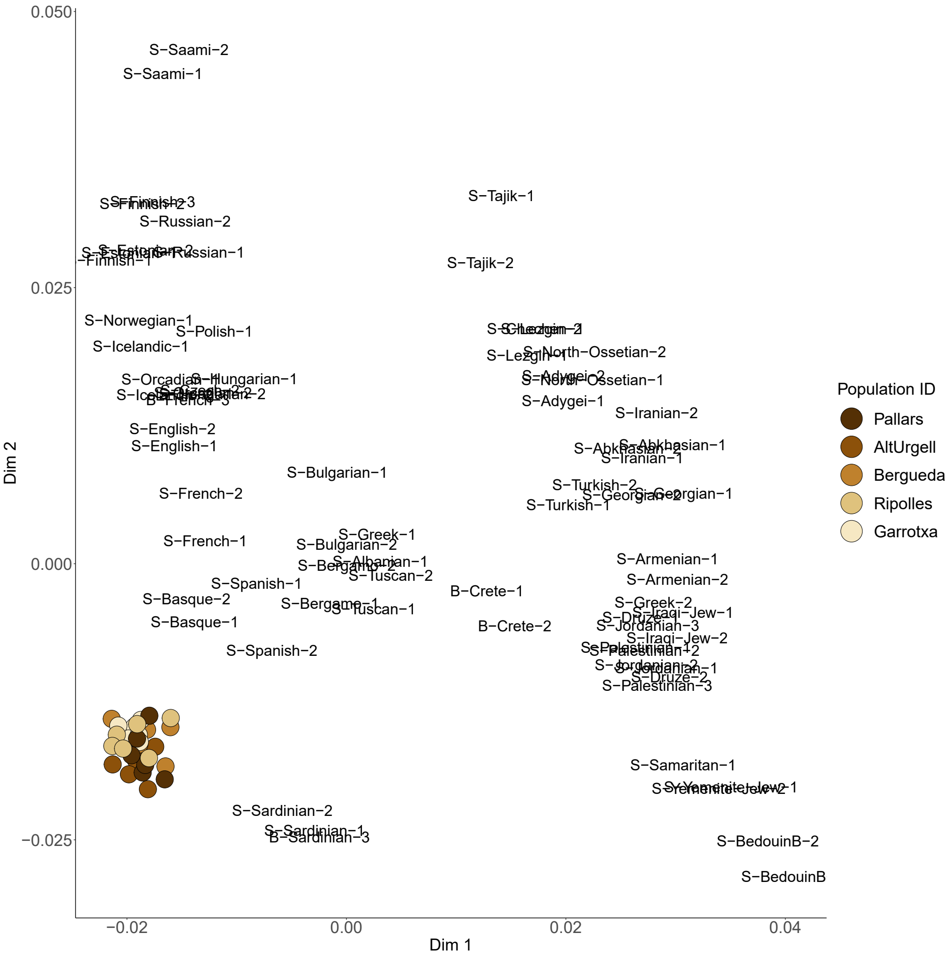

Supplementary Figure 2. Multidimensional scaling using SEP samples (circles, colours denote origin) and SGDP West Eurasian samples.

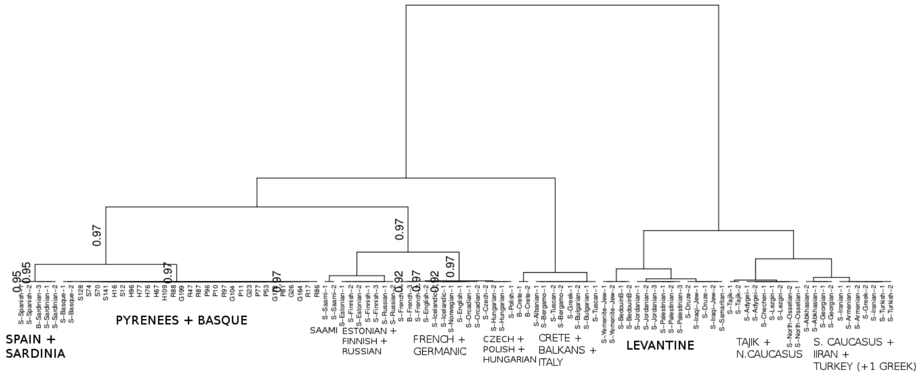

Supplementary Figure 3. fineSTRUCTURE tree showing the relationship of SEP regions with other West-Eurasian populations using data from the SEP-SGDP dataset.

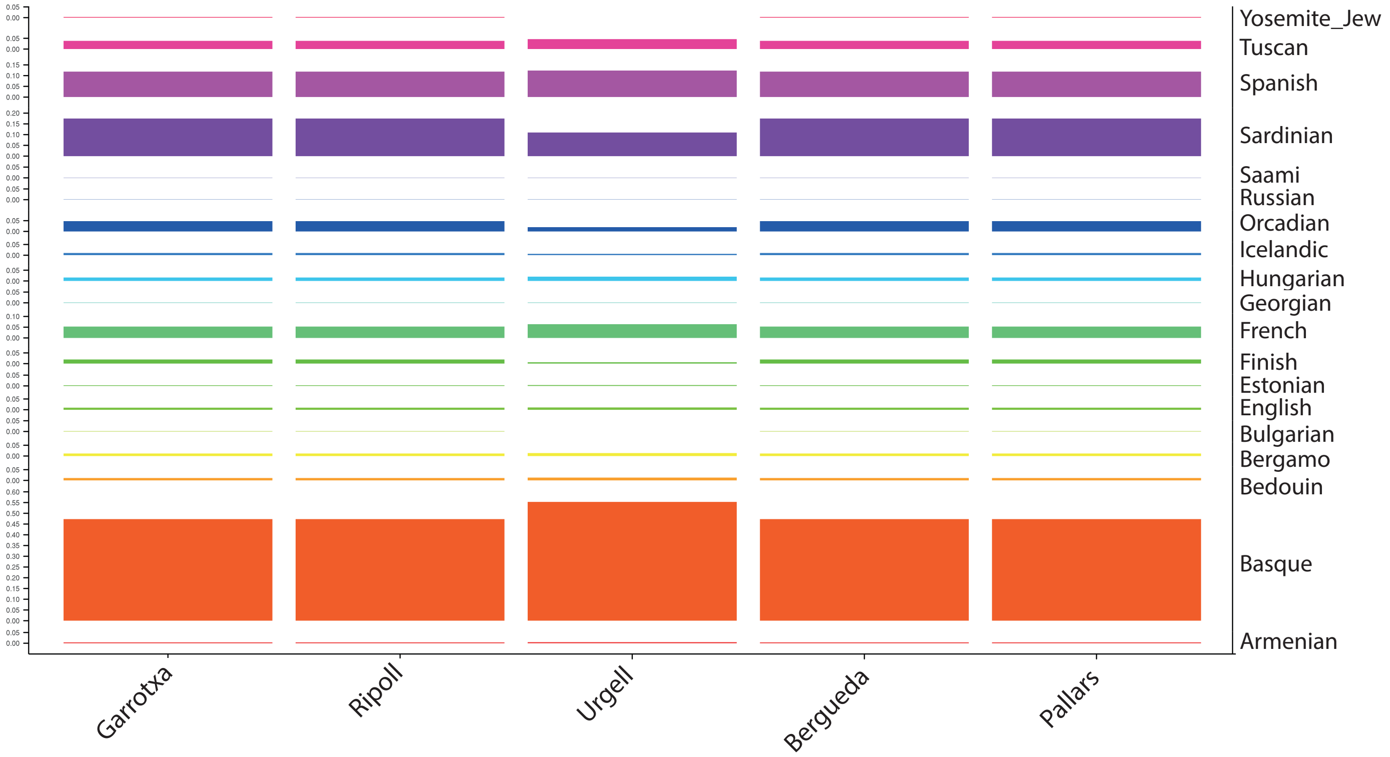

Supplementary Figure 4. GLOBETROTTER ancestral components plot showing the SGDP populations (y-axis) that contributed to the haplotype profiles of the recipient SEP populations (x-axis).

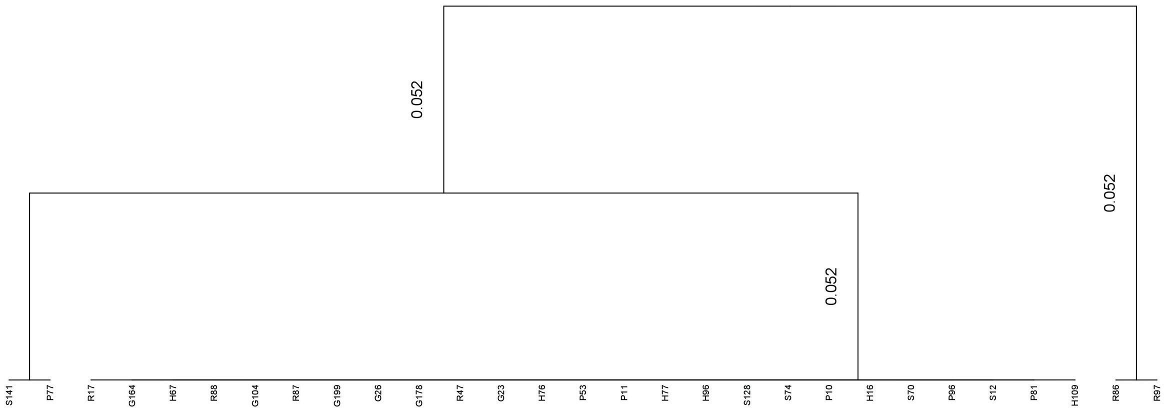

Supplementary Figure 5. fineSTRUCTURE tree including SEP samples and based on 231K SNPs that are common with the Biagini et al. 2019 dataset and present variation in more than one sample. Note that the downsampling causes the patterns of population structure -observed when the full amount of variation is included in the analyses- to disappear. SEP sample codes are explained in the Supplementary table.

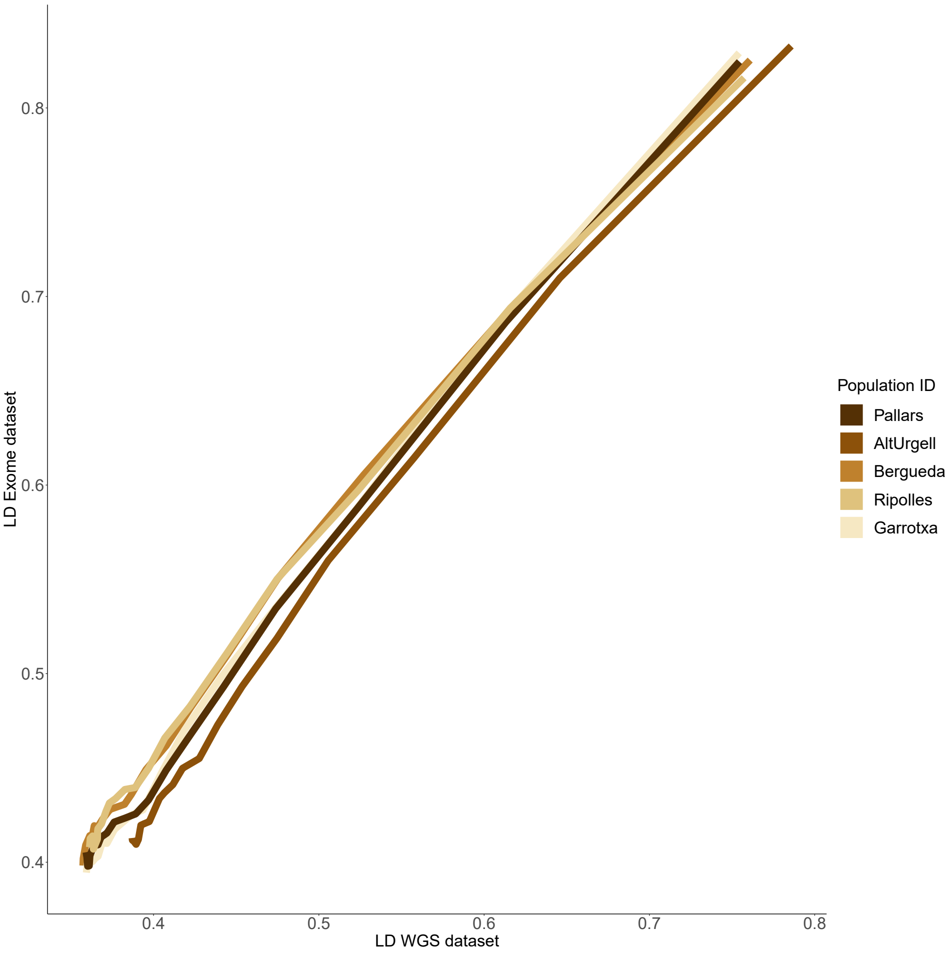

Supplementary Figure 6. Comparison between the results of the HR algorithm regarding LD between WGS and Exome datasets in SEP samples. In the x-axis is represented the HR score in the WGS dataset, in the y-axis is represented the HR score in the exome dataset.

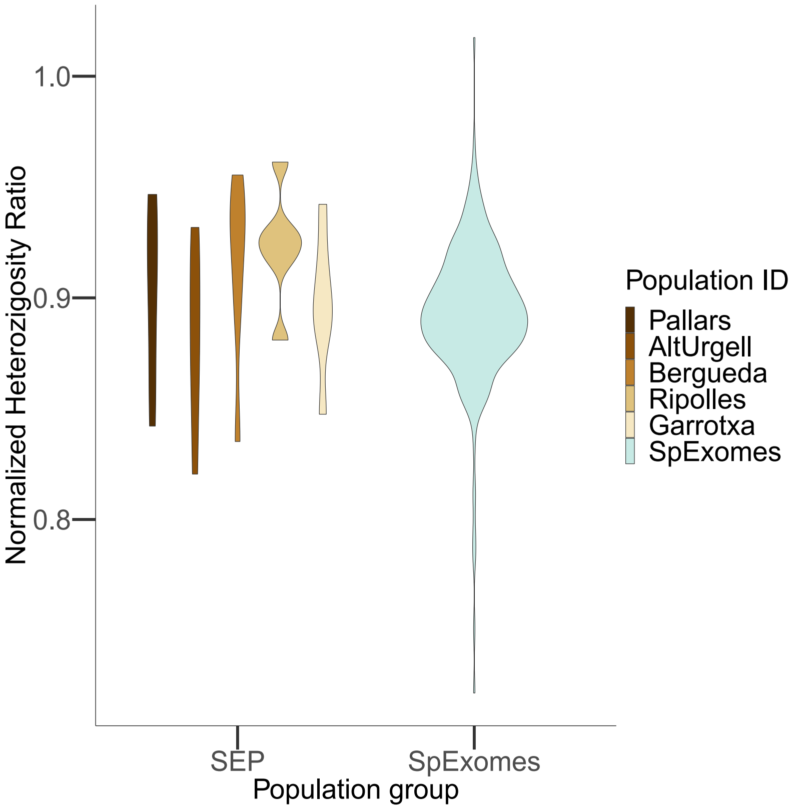

Supplementary Figure 7. Normalized Heterozigosity Ratio of SEP and SpExomes samples. SEP samples nHetR fall inside the distribution of the score for SpExomes samples.

Supplementary information

Fine-scale population structure in five rural populations from the Spanish Eastern Pyrenees using high-coverage whole-genome sequence data

Iago Maceda, Miguel Martin Álvarez, Georgios Athanasiadis, Raúl Tonda, Jordi Camps, Sergi Beltran, Agustí Camps, Jordi Fàbrega, Josefina Felisart, Joan Grané, José Luis Remón, Jordi Serra, Pedro Moral, and Oscar Lao

SEP WGS data cleaning

Data cleaning was performed using a strict threshold of 0% missingness and excluding those SNPs that were out of HWE. As a last step for data cleaning, we checked for kinship between our individuals using KING (1), resulting in the exclusion of one individual from Alt Urgell. Only the autosomes were kept at the end of the data cleaning step. The final dataset contained 29 individuals and 9,309,056 biallelic polymorphic SNPs.

Inference of the ancestral allele

In order to define the derived allele needed in the ABC-DL algorithm, we downloaded the best reciprocal alignments between Hg19 and PanTro4 from the UCSC in AXT format. From these files we reconstructed a chimpanzee genome adding "-" (no base) as filler for the gaps in the alignment, using chromosome lengths from Hg19 from the UCSC. An intermediate BED was generated containing the chimpanzee alleles for the SNPs present in the VCF, which was merged with the original VCF. Those SNPs for which the chimpanzee allele was either unknown or not properly aligned were filtered out.

Identification of genetic barriers and anisotropy patterns in the SEP dataset using the shared coancestry matrix

For samples from the same geographic group, one can define the minimum genetic distance expected (nugget, *n*), as well as a maximum genetic distance no matter how much geographically distant are the two samples (sill, *s*). If the genetic differentiation follows a direction in space (i.e. there is a genetic gradient in the data points), then the model can be extended to include an angle α of maximum genetic differentiation over space. We define the genetic distance between two points as:

$$(1) \quad GenDist = s * k + n$$

$$(2) \quad k = \frac{\sqrt{b^2 * (x * \cos(\alpha) + y * \sin(\alpha))^2 + \ a^2 * (x * \sin(\alpha) - y * \cos(\alpha))^2}}{a^2 * b^2}$$

In particular, under the Multiple regressions on distance matrices (2) framework, a and b can be estimated using classical nonlinear regression fitting methods. Here we applied the Nelder and Mead Simplex method implemented at Flanagan's JAVA package (3). For a given K geographic groups, the identification of the genetic barriers consists on identifying the set of geographically related points that minimizes the goodness of fit of the estimated parameters. Let be the mean sum of square error of each point between the observed genetic distance and the inferred D' from the fitted parameters:

$$(3)\ SSE = \sum_{g}^{K} SquareError_g = \begin{cases} \sum_{i=1}^{n_g}\sum_{j=1}^{n_g} \frac{\left(D(i,j) - D'(i,j)\right)^2}{m-1} & if\ \frac{n_g(n_g-1)}{2} \geq 3 \\ \sum_{i=1}^{n_g}\sum_{j=1}^{n_g} \frac{(D(i,j) - \bar{D})^2}{m-1} & if\ \frac{n_g(n_g-1)}{2} < 3 \end{cases}$$

In order to optimize the SSE function and to identify the genetic barriers, we define each geographic group by a [x,y] pair of coordinates, and assign each observed point based on its proximity to each geographic group, such as in K-means algorithm. The problem is then to find the geographic coordinates of each group that minimize the SSE. In order to explore the space of possible solutions, we propose using a genetic algorithm. This type of approaches have been already applied in optimization problems involving geographic divisions (4).

Estimation of the effective population sizes and time of split of SEP populations

A total of 300,000 simulations were generated using *fastsimcoal2* (5), each simulating 7,314 genomic regions separated by at least 100 kb encompassing ~647 Megabases (Mb), that do not contain CpG islands or genes. The generation time was 29 years (6), the mutation rate was set to 1.61e-8 with a standard deviation of 0.13e-8 (7), and a migration rate between the populations was sampled from a uniform distribution from 5e-4 to 5e-3. From the 300,000 simulations, a total of 30,000 simulations were used in the DL training, and the remaining 270,000 in the ABC step. To generate the *jSFS* we used one sample from each region. We added noise to the *jSFS* before merging it with the simulation. This merged dataset (sample *jSFS* and the simulation) was employed in the artificial neural network (ANN) training (see (8) for details of the implementation). For each parameter of the demographic model, a total of 10 independent ANN's, each featuring four neural layers with 100 neurons, were trained using resilient backpropagation and dropout at 0.1 for a maximum duration of 2.5 hours, or until an error <0.01 was reached. In order to generate a single summary statistic out of all the 10 independent ANN's, we estimated the Spearman correlation between the predicted value by each ANN and the value of the parameter used in the simulation and ascertained the ANN that provided a better Spearman correlation.

For the ABC step, the remaining individuals not used in the noise injection step were considered. We generated 50 resampled datasets by taking one individual at random from each population. The final *jSFS* was estimated averaging over all the sampled datasets.

This final *jSFS* profile was used in the trained ANN's to predict the different parameters. We used the *abc* package (9) with the local linear regression algorithm (10) and logit transformation (11) to estimate the posterior distributions of each parameter. For each posterior distribution, we estimated the mean, median and half range mode using genefilter (12) as centrality statistics, and the 95% Credible Interval and the High Density Interval computed in bayestestR (13) as dispersion statistics.

Supplementary Information References

1. Manichaikul A, Mychaleckyj JC, Rich SS, Daly K, Sale M, Chen WM. Robust relationship inference in genome-wide association studies. Bioinformatics. 2010 Nov 15;26(22):2867–73.
2. Legendre P, Legendre L. Numerical Ecology. 3rd Editio. Elsevier Ltd; 2012.
3. Thomas-Flanagan M. Regression Class: Linear and Non-linear Regression. 2016.
4. Sergeeva M, Delahaye D, Mancel C, Vidosavljevic A. Dynamic airspace configuration by genetic algorithm. J Traffic Transp Eng (English Ed. 2017 Jun 1;4(3):300–14.
5. Excoffier L, Dupanloup I, Huerta-Sánchez E, Sousa VC, Foll M. Robust Demographic Inference from Genomic and SNP Data. PLoS Genet. 2013 Oct;9(10):1003905.
6. Fenner JN. Cross-cultural estimation of the human generation interval for use in genetics-based population divergence studies. Am J Phys Anthropol. 2005 Oct;128(2):415–23.
7. Lipson M, Loh P-R, Sankararaman S, Patterson N, Berger B, Reich D. Calibrating the Human Mutation Rate via Ancestral Recombination Density in Diploid Genomes. Coop G, editor. PLOS Genet. 2015 Nov 12;11(11):e1005550.
8. Mondal M, Bertranpetit J, Lao O. Approximate Bayesian computation with deep learning supports a third archaic introgression in Asia and Oceania. Nat Commun. 2019 Dec 16;10(1):246.
9. Csilléry K, François O, Blum MGB. abc: an R package for approximate Bayesian computation (ABC). Methods Ecol Evol. 2012 Jun;3(3):475–9.
10. Beaumont MA, Zhang W, Balding DJ. Approximate Bayesian Computation in Population Genetics. Genetics. 2002;162(4):2025–35.
11. Fagundes NJR, Ray N, Beaumont M, Neuenschwander S, Salzano FM, Bonatto SL, et al. Statistical evaluation of alternative models of human evolution. Proc Natl Acad Sci U S A. 2007;104(45):17614–9.
12. Gentleman R, Carey V, Huber W, Hahne F. genefilter: methods for filtering genes from high-throughput experiments. 2019.
13. Makowski D, Ben-Shachar M, Lüdecke D. bayestestR: Describing Effects and their Uncertainty, Existence and Significance within the Bayesian Framework. J Open Source Softw. 2019 Aug 13;4(40):1541.
